# The Effect of Parental Education and Socioeconomic Status on Dental Caries among Saudi Children

**DOI:** 10.3390/ijerph182211862

**Published:** 2021-11-12

**Authors:** Passent Ellakany, Marwa Madi, Shaimaa M. Fouda, Maria Ibrahim, Jehan AlHumaid

**Affiliations:** 1 Department of Substitutive Dental Sciences, College of Dentistry, Imam Abdulrahman Bin Faisal University, Dammam 31441, Saudi Arabia; smfouda@iau.edu.sa; 2 Department of Preventive Dental Sciences, College of Dentistry, Imam Abdulrahman Bin Faisal University, Dammam 31441, Saudi Arabia; mimadi@iau.edu.sa (M.M.); msIbrahim@iau.edu.sa (M.I.); jaalhumaid@iau.edu.sa (J.A.)

**Keywords:** caries, parental education, income, medical insurance, teeth brushing, fluoridated toothpaste

## Abstract

Parental attitudes toward the importance of oral hygiene have an impact on the formation of their children’s oral habits and the prevalence of oral diseases. Our aim was to assess the association between parents’ education and socioeconomic status and their children’s oral health. A cross-sectional study was conducted between the years of 2018 and 2020 in the eastern province of Saudi Arabia among primary school children. Two pre-calibrated dentists performed the clinical examination of the children, and a self-administered validated questionnaire was obtained from their parents. Clinical examination was performed on 589 children with an age range of 3 to 14 years, where 47% were males and 53% were females, 70% with dental caries. Both parents with higher education and a high monthly income were significantly associated with lower prevalence of decayed teeth in their children, respectively. Mother’s education, age, gender and application of sealant were found significantly associated with the high prevalence of caries. High prevalence of tooth decay was reported among school children in the eastern province of Saudi Arabia. A high educational level of parents and high income were correlated with a lower prevalence of decayed teeth, similarly to the situation in the case of presence of medical insurance.

## 1. Introduction

Dental caries is one of the major oral health problems affecting more than 60% of schoolchildren [1]. It causes several complications that lead to over-priced and time-consuming dental treatment [2,3]. Numerous studies conducted in different countries showed that applying preventive measures and improving the social environment significantly reduce the frequency of dental caries [4,5].

Socio-economic status is directly reflected in the eating habits and the lifestyle patterns which affect the prevalence of dental caries [6,7]. Moreover, gender differences in dietary behavior have been reported where boys tend to eat sweets and drink soft drinks more often than girls do [8].

Many epidemiological studies have observed that the rate of dental caries was effectively controlled by enhancing the optimum oral hygiene preventive protocol [9,10,11,12,13]. Parental attitudes toward the importance of oral hygiene play a major role in the preservation of healthy children’s teeth. Parents create the necessary environment for a healthy lifestyle, improve children’s self-confidence, and assist in habit formation [10]. Parental skills and attitudes toward oral hygiene may have a significant impact on the development of their children’s oral hygiene habits, the prevalence of oral diseases, controlling their children’s tooth brushing, and sugar-snacking habits, which represents the most significant predictor of children’s favorable habits [11]. Moreover, studies have reported that parental education and family income have a direct effect on children’s oral health [10,12].

Parents’ belief in the importance of oral health for overall health is as a major factor for seeking professional preventive dental care. However, several structural barriers such as transportation, school absence policies, and difficulty locating providers also affect seeking dental care [13]. Perceived barriers among parent attitudes are likely important [14]. Low family income and fearing the costs of dental treatments, inability to pay, lack of oral health insurance, and inadequate insurance coverage are strong barriers to receiving dental services. Some parents believe that children did not seek dental treatment unless they were in pain. Others avoided seeking dental treatment because of fear or anxiety either on their own or for their children due to previous negative dental experiences [15].

Children’s adoption of consistent behavioral habits begins at home with their parents, especially mothers; they have a great influence on the child’s oral health behavior [10]. Children’s oral cavity occurrence is greatly affected by a frequent tooth-brushing habit and a healthy diet, which prevents the presence of caries biofilm which is the principal factor of developing caries in the presence of fermentable sugars and carbohydrates and might result in teeth demineralization. In some cases, especially in those with diabetes mellitus, the total amount of sugar is reduced but dental caries exists as a result of the presence of cariogenic biofilm [16].

Compared with their higher-income counterparts, children growing up in low-income families were reported to complete less schooling and achieve lower results, report worse health, and both work and earn less in adulthood [17].

Limited data are available about the association between parents’ socioeconomic status and children’s oral health in Saudi Arabia. The aim of the present study was to evaluate the effect of parental education and income on their children’s oral health. The study hypothesis stated that there is a correlation between high parent education and income and improved child oral health.

## 2. Materials and Methods

### 2.1. Study Population and Design

A cross-sectional study was conducted in Saudi Arabia starting from December 2019 till January 2020. A paper-based self-administered questionnaire was submitted to 660 participants. The study was approved by the College of Dentistry and the Institutional review board of Imam Abdulrahman Bin Faisal University (IRB-2017-02-048) following the STROBE (Strengthening the Reporting of Observational studies in Epidemiology) guidelines. A total of 589 Saudi parents of primary school-aged, healthy children with an age range of 3 to 14 years were included in the study and accepted the clinical examination of their children. School children with any systemic diseases or health disorders and siblings of the same family were excluded.

The dental examination was performed by two precalibrated trained dentists, using a WHO periodontal probe under natural light and a standard size 4 mirror, following the WHO recommendations for oral health surveys [18]. The number of decayed, restored teeth, presence of fissure sealant, presence of malocclusion, and gingivitis were recorded for each examined child.

Caries was defined as a cavitated lesion. Dental plaque index was monitored visually with the help of the WHO CPI probe to determine the presence/absence of dental plaque on the buccal surfaces of the explored teeth number # 16, 11, 26, 36, 31, and 46 to assess gingivitis.

The parents were asked to answer the questionnaire including the following sections: the first section was composed of social and demographical characteristics such as: name, age of the child, parental approval to examine their children’s oral health, acceptance of topical fluoride application, and approval of photographing their children in addition to their signatures.

The second section included questions about the children’s past dental history, such as presence of dental pain and the last dental clinical appointment in the previous 6 months, mentioning the causes of this dental visit. They were asked to state the obstacles they might have faced in reaching proper dental treatment for their children and mention the availability of dental insurance or not. Moreover, they were questioned about their children’s performance in tooth brushing, the use of fluoridated toothpastes and the quantity of junk food intake.

Finally, the third section was composed of questions about the educational level of both parents and the family monthly income. The responses were given in the form of yes, no or don’t know/remember in the questionnaire.

### 2.2. Statistical Analysis

The exposure variables in the current study were social and demographical qualities (child age, gender, father’s educational level, mother’s educational level, family monthly income, and the availability of medical insurance) and the child’s oral behavior (tooth brushing, use of fluoridated toothpaste, sealant application) while the outcome variables were the child’s oral hygiene status (presence of caries, gingivitis, and malocclusion). Descriptive statistics were calculated as frequencies, percentages, means and standard deviations. Chi-square or Fisher’s exact test were performed to assess the relationship between exposure and outcome variables where appropriate. Significance was set at *p* value < 0.05. Multivariate logistic regression analysis was performed and adjusted odds ratio (OR) and 95% confidence interval (CL) were calculated and presented in table. Data were analyzed using SPSS statistical software for Windows version 23.

## 3. Results

Demographic characteristics of participants showed that the mean age of the children was 7.48 ± 1.57 (ranged 3–14) years old. Clinical examination was performed on 589 (89%) children after obtaining signed informed consent from their parents. Out of the children, 53% were female and remaining 47% were male. Prevalence of caries was recorded as 70%, followed by the presence of gingivitis in 93 (16%) children, and malocclusion was recorded in only 85 (14%) children. Table 1 shows all the demographical characteristics of these children. Mostly mothers had a university-level degree, with an 7000 SAR to 20,000 SAR family monthly income. Almost all children had excellent school performance (76%) with no learning difficulties (42%). Only 5% of children received sealant application on permanent molars.

Table 2 represents the comparison between caries prevalence and demographic characteristics. Education level of parents was significantly associated with caries presence; fathers with low education (50%) and mothers with higher education (49%) were similarly significantly associated with caries prevalence (0.0001, 0.004, respectively). Higher caries prevalence was found among the middle age-grouped children (6–9 years, 62%) and the association between age and caries presence was statistically significant (0.0001). Usage of fluoridated toothpaste was also significantly associated with caries presence (41%). Middle class family income and no insurance were also significantly associated with the high prevalence of caries (52% and 51%, *p* = 0.0001, 0.035, respectively).

Univariate and multivariate analyses were conducted for testing the association of demographical characteristics with the presence of caries. All variables showing significant association with the caries in univariate analysis were used together in multivariate regression to present the model, and results from multivariate regression are presented in Table 3. Mothers with a university-level degree were significantly associated with the high prevalence of dental caries (OR = 0.5, 95% CL = 0.23−1.00) (*p* = 0.050). Elder participants had higher prevalence compared to the youngest age group in the study (3–5 years). The eldest participants (10–14 years) were found with the highest prevalence (OR = 3.22, 95% CL = 1.58–6.6) (*p* = 0.001). Female participants were found with a higher prevalence than male (OR = 3.63, 95% CL = 1.05−12.6). The brushing habit and fluoridated toothpaste showed no significant association.

## 4. Discussion

Tooth decay could adversely affect children’s health, quality of life, psychosocial well-being, and education [19,20]. The present study evaluated the effect of parental education and income on their children’s oral health.

The results showed a high prevalence (70%) of tooth decay among children. Similar findings were reported in previous studies among gulf countries [21,22,23,24]. This might explain the high incidence of dental caries disease among the community despite an improvement in educational and financial status of citizens and elaborating the need for applying oral health awareness programs among parents and children [25].

Additionally, only 11% of the parents reported that their children brush their teeth and 26% reported the use of fluoridated toothpaste. However, no significant association between both factors or independently and the prevalence of dental caries was observed. This may reflect other tooth brushing-related factors, such as the frequency, amount and duration of tooth brushing, that have a greater effect on tooth decay rather than the type of toothpaste used alone [26,27]. Additionally, the use of fluoridated toothpaste was not sufficient to prevent tooth decay, rather than the parents’ awareness of their children brushing habits. This is in agreement with previous studies that showed an enhanced anti-cariogenic effect of fluoridated toothpastes when compared to a placebo in supervising tooth brushing procedure [28,29]. Thus, parents need to be aware about the major role of brushing habits and soft drink intake in dental caries progression and prevention.

The study hypothesis was partially rejected as the results showed that parents with higher educational level and income were associated with lower prevalence of dental caries. In line with our findings, previous studies reported reduced prevalence of dental caries and improved oral health of children whose parents had high education and income [30,31,32]. This could be explained by the lack of knowledge about the importance of oral hygiene and preventive measures among less educated parents in comparison to higher ones [33,34].

In studying the role of each parent independently, it was found that higher maternal education was significantly associated with higher caries prevalence, unlike father’s educational impact, which agrees with other studies [35,36]. This might be due to the employment status of mothers who would not have enough time to develop proper oral hygiene and health dietary habits regime with their children [37]. An alternative explanation might be that highly educated mothers with a high socioeconomic level in KSA are used to intaking meals including more soda and sugar-sweetened beverages [38] and fast food [39]. However, specific data on the employment status and the dietary composition of family meals were not collected in the current study. On the other hand, educated mothers can easily detect the suitable healthy lifestyle by merging sports with healthy food when weight gain is noticed among their children [33,40]. The socioeconomic status affects the eating habits, which in turn are correlated with caries prevalence, particularly the high sugar intake [34,41].

Our findings showed that age was highly linked to caries prevalence where elder children exhibited more caries than younger ones. This is in agreement with Youssefi et al.’s [42] study, where a lack of knowledge about the methods of oral healthcare towards primary teeth may be the causative factor for higher caries prevalence, as is also parental belief regarding the importance of retaining the primary teeth in the oral cavity as they are exchangeable with permanent teeth so they are of minimal importance [43,44,45], since early shedding of primary teeth would expose permanent teeth early to cariogenic factors [46,47,48,49,50]. Additionally, anxiety and fear from dental treatment was seen increasing in older age [51].

Gender also showed significant association, where females were more prone to caries in the current study than males, which is in a line with several other studies [52,53]. Girls usually exhibit earlier eruption of teeth than boys which subject them to higher caries incidence [54]. Additionally, dental fear was reported to be higher among girls than boys, which might hinder girls from going to dental visits and performing needed dental treatment [55,56]. On the other hand, boys in the Arabic culture are not raised to express their own fears as this represents a point of weakness on their personalities if they did [51].

The present study showed that lack of medical insurance had a significant effect on the prevalence of decayed/caries teeth. The reason for that could be financial problem due to the high cost of dental treatments, living in rural areas away from pediatric dentists with difficulty in transportation [57,58], where dental treatment exhibits a higher financial barrier than medical treatment [59].

Children’s oral health requires more attention by developing more public preventive healthcare programs that would serve children and reduce the incidence of caries [25]. Parental dental education is also of great importance in order to provide their children with healthy dietary products and perform frequent dental follow ups according to the scheduled dental visits. Therefore, prevention of dental problems could have a positive impact on the children’s education and quality of life [20].

### Strengths and Limitations

The oral health of school children was determined after clinical examination by calibrated dentists. The eastern province is the largest province by area in Saudi Arabia with the third largest population. However, the findings of the current study cannot be generalized to the whole kingdom and are not comparable to rural areas that might not have the same dental care services available as in the urban regions. Additional studies are needed to assess the oral hygiene of elder students in different areas of Saudi Arabia. Further analysis of the children eating habits as well as the frequency of soft drinks intake needs to be conducted through implementing a diet questionnaire. The study is limited by its cross-sectional nature and lack of data about parents’ oral health knowledge and attitude.

## 5. Conclusions

A high prevalence of tooth decay was reported among school children in the eastern province of Saudi Arabia. Parental education, age and gender status were key factors for the high prevalence of dental caries among children. Socioeconomic status is affected by various factors, including family income, parents’ educational background, their inherited health beliefs, traditional eating habits and their knowledge about a healthy lifestyle. A high educational level of parents and high income were correlated with a lower prevalence of decayed teeth, so it was unlikely that the situation was due to a lack of medical insurance.

## Figures and Tables

**Table 1:** Sample Characteristics.

Age (N = 589)	Minimum	3
Year	Maximum	14
	Mean ± SD	7.48 ± 1.57
Gender (N = 589) n (%)	Male	274 (46.52)
	Female	315 (53.48)
Caries Status (N = 589) n (%)	No Caries	176 (29.88)
	Caries	413 (70.12)
Gingival Status (N = 589) n (%)	No Gingivitis	496 (84.21)
	Gingivitis	93 (15.79)
Occlusion Status (N = 589) n (%)	No Malocclusion	504 (85.57)
	Malocclusion	85 (14.43)
Father Educational Level (N = 589) n (%)	Intermediate School Level or Less	267 (45.33)
	High School Level	126 (21.39)
	University Level	196 (33.28)
Mother Educational Level (N = 589) n (%)	Intermediate School Level or Less	71 (12.05)
	High School Level	205 (34.80)
	University Level	313 (53.14)
Family Monthly Income (N = 589) n (%)	Less than 7000 SR	150 (25.47)
	7000–20,000 SR	314 (53.31)
	More than 20,000 SR	122 (20.82)
Sealants Status (N = 589) n (%)	Sealant	27 (4.58)
	No Sealant	562 (95.42)
Use Fluoridated Toothpaste (N = 589) n (%)	Yes	154 (26.15)
	No	262 (44.48)
	I don’t know	173 (29.37)
Brushing Habits (N = 588) n (%)	Yes	64 (10.88)
	No	205 (34.86)
	I don’t know	319 (54.25)

**Table 2:** Comparisons of caries with demographical variables.

Variable	No Caries	Caries	*p*-Value
	n (%)	n (%)	
Mother Educational Level			
Intermediate Level and Below	11 (6)	60 (15)	0.004
High School Level	56 (32)	149 (36)	
University Level	109 (62)	204 (49)	
Father Educational Level			
Intermediate Level and Below	59 (34)	208 (50)	0.000
High School Level	41 (23)	85 (21)	
University Level	76 (43)	120 (29)	
Age			
3–5 Years	61 (35)	68 (16)	0.000
6–7 Years	52 (30)	110 (27)	
8–9 Years	40 (23)	144 (35)	
10–14 Years	23 (13)	91 (22)	
Gender			
Male	98 (56)	176 (43)	0.004
Female	78 (44)	237 (57)	
Existing Sealant			
No	173 (98)	389 (94)	0.029
Yes	3 (2)	24 (6)	
Brushing Habits			
No	14 (8)	50 (12)	0.219
Yes	68 (39)	137 (33)	
I don’t know	94 (53)	225 (54)	
Fluoridated Toothpaste			
No	37 (21)	117 (28)	0.038
Yes	92 (52)	170 (41)	
I don’t know	47 (27)	126 (31)	
Income			
Below SAR 7000	27 (15)	123 (30)	0.000
SAR 7000 to SAR 20,000	101 (57)	213 (52)	
More than SAR 20,000	48 (27)	74 (18)	
Type of Dental Clinics			
Private Clinics	115 (65)	252 (61)	0.338
Governmental Clinics	61 (35)	160 (39)	
Insurance			
No	60 (34)	175 (42)	0.035
Yes	108 (61)	211 (51)	

**Table 3:** Multivariate regression analyses for caries odds ratio adjusted for multiple variables.

Covariate	Odds Ratio	95% Conf. Interval		*p*-Value
Mother Educational Level				
Intermediate Level and Below	(Ref)	(Ref)		(Ref)
High School Level	0.6953948	0.3260412	1.483168	0.347
University Level	0.4794515	0.229693	1.000787	0.050 *
Father Educational Level				
Intermediate Level and Below	(Ref)	(Ref)		(Ref)
High School Level	1.01674	0.5567707	1.856706	0.957
University Level	0.9692784	0.5586719	1.681668	0.912
Age				
3–5 Years	(Ref)	(Ref)		(Ref)
6–7 Years	1.751673	1.01458	3.024265	0.044 *
8–9 Years	3.166034	1.780199	5.630703	0.000 *
10–14 Years	3.228422	1.575964	6.613545	0.001 *
Gender				
Male	(Ref)	(Ref)		(Ref)
Female	1.54824	1.033485	2.319382	0.034 *
Existing Sealant				
No	(Ref)	(Ref)		(Ref)
Yes	3.639601	1.051623	12.59643	0.041 *
Brushing Habits				
No	(Ref)	(Ref)		(Ref)
Yes	0.6684471	0.331933	1.34612	0.259
Don’t know	0.5654504	0.2873434	1.112725	0.099
Fluoridated Toothpaste				
No	(Ref)	(Ref)		(Ref)
Yes	0.8344126	0.502455	1.385685	0.484
I don’t know	0.7412031	0.4356856	1.26096	0.269

*: statistically significant at *p* < 0.05.

## Data Availability

Data are available upon request from corresponding author.
